# Bilateral carcinoid heart disease secondary to a massive right-to-left shunt through a patent foramen ovale: a case report

**DOI:** 10.1093/ehjcr/ytaf444

**Published:** 2025-09-16

**Authors:** Javier Bertolín Boronat, Begoña Muñoz Giner, María Teresa Tuzón Segarra, David Tejada Ponce, Juan Cosin-Sales

**Affiliations:** Cardiology Department, Arnau de Vilanova Hospital, Carrer de Sant Clement 12, Campanar, 46015 Valencia, Spain; Cardiology Department, Arnau de Vilanova Hospital, Carrer de Sant Clement 12, Campanar, 46015 Valencia, Spain; Cardiology Department, Arnau de Vilanova Hospital, Carrer de Sant Clement 12, Campanar, 46015 Valencia, Spain; Cardiology Department, Arnau de Vilanova Hospital, Carrer de Sant Clement 12, Campanar, 46015 Valencia, Spain; Cardiology Department, Arnau de Vilanova Hospital, Carrer de Sant Clement 12, Campanar, 46015 Valencia, Spain

**Keywords:** Case report, Carcinoid syndrome, Carcinoid heart disease, Patent foramen ovale, Shunt

## Abstract

**Background:**

Carcinoid heart disease (CHD) is a rare complication of neuroendocrine tumours (NET). It typically involves the right-sided heart valves due to the effect of vasoactive substances. Bilateral involvement is uncommon and usually requires a significant right-to-left shunt through a patent foramen ovale (PFO).

**Case summary:**

A 75-year-old woman with metastatic well-differentiated NET presented with progressive dyspnoea. Transthoracic echocardiography showed multivalvular involvement, including the tricuspid, mitral, and aortic valves, secondary to CHD. During preoperative assessment for valve replacement, the patient developed refractory hypoxaemia. A large right-to-left shunt through a high-risk PFO was confirmed by transoesophageal echocardiography. Percutaneous PFO closure was performed, which resulted in immediate normalization of oxygen saturation. Unfortunately, a few days later, the patient developed aspiration pneumonia and died of cardiorespiratory arrest.

**Discussion:**

To our knowledge, this is the first reported case of bilateral CHD due to a massive right-to-left shunt through a PFO, driven by elevated right atrial pressure and a tricuspid regurgitation jet. This case highlights the importance of assessing for a PFO in patients with carcinoid syndrome and left-sided valvular involvement or unexplained hypoxia. Early recognition and PFO closure may represent a key therapeutic step in selected patients.

Learning points

- Agitated saline contrast echocardiography should be performed to detect patency of the foramen ovale in patients with carcinoid heart disease.
- Patent foramen ovale closure should be considered as early as possible in patients with bilateral carcinoid heart disease and hypoxaemia.

## Introduction

Neuroendocrine tumours (NETs) are uncommon neoplasms that secrete vasoactive substances, mainly serotonin, which are responsible for carcinoid syndrome (CS).^1,2^ These substances are usually inactivated by the liver and lungs, preventing systemic effects and carcinoid heart disease (CHD) in most patients.^3^ The symptoms of CS typically include gastrointestinal hypermotility, cutaneous flushing, and bronchospasm.

CHD is characterized by fibrous thickening of the endocardium, most commonly affecting the right-sided valves and potentially leading to heart failure. The tricuspid valve is the most frequently affected.

Bilateral CHD is rare and may develop when both hepatic and pulmonary clearance are bypassed. This can occur in the presence of hepatic metastases and a right-to-left shunt through a patent foramen ovale (PFO), which allows vasoactive substances to reach both sides of the heart.

We report an exceptional case of bilateral CHD and a large right-to-left shunt through a PFO. The shunt PFO, rather than the valvular disease, proved to be the main issue in this patient.

## Summary figure

**Figure:**
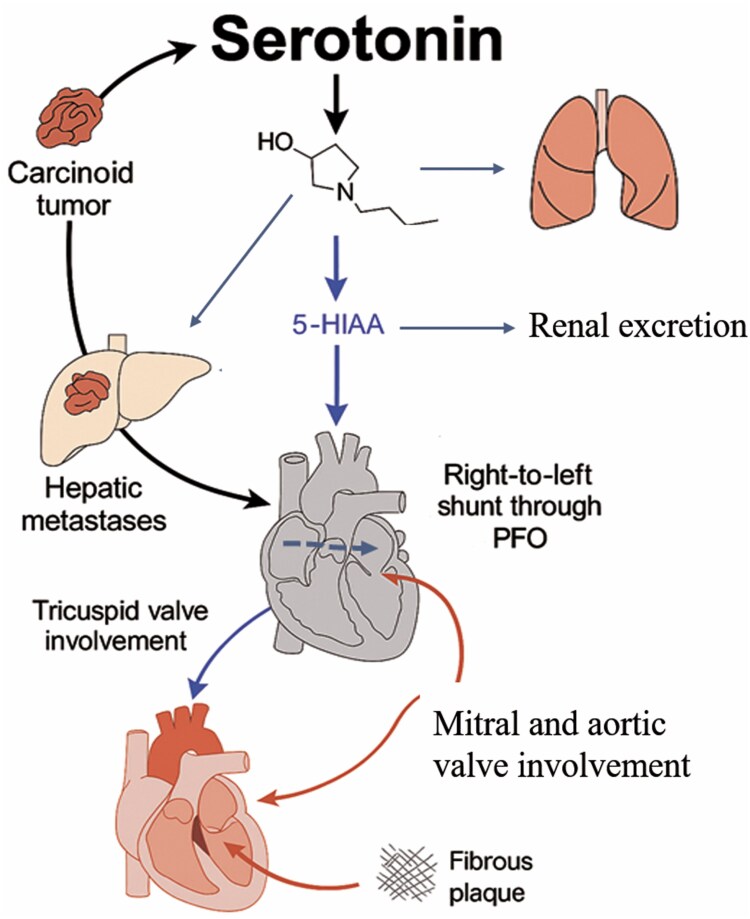


## Case presentation

A 75-year-old woman with no cardiovascular risk factors initially presented with constitutional symptoms in 2018, characterized by anorexia and weight loss. An outpatient blood test showed an increase in hepatic transaminases, chromogranin A and 5-hydroxyindoleacetic acid levels, raising suspicion of a neuroendocrine tumour. Subsequent abdominal ultrasound and CT scan results indicated the presence of multiple hepatic parenchymal lesions. A liver biopsy confirmed a well-differentiated, low-grade neuroendocrine tumour with hepatic metastases. The patient started chemotherapy, including monthly Sandostatin by symptoms consistent with CS.

**Figure 1:**
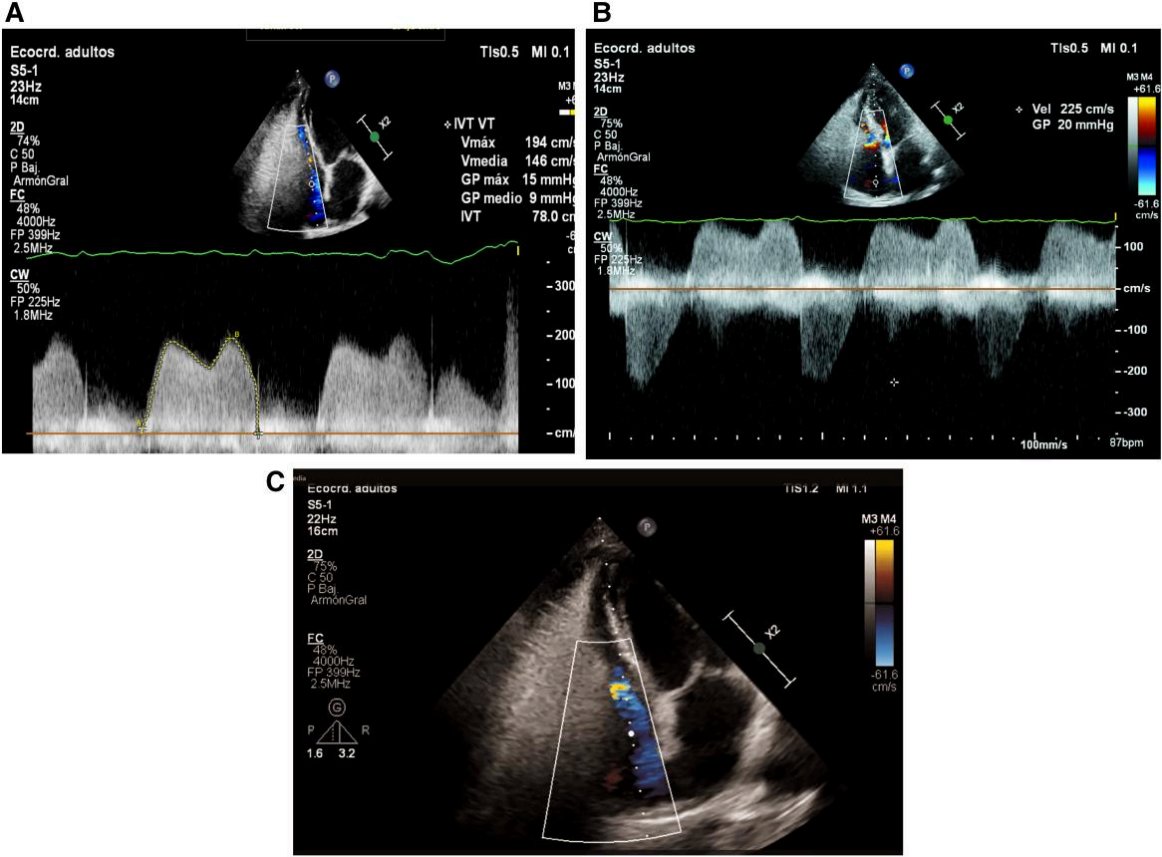
Mean gradient across the tricuspid valve (MG: 9 mmHg), consistent with severe tricuspid stenosis (*A*). Continuous-wave Doppler showing tricuspid regurgitation (*B*). Doppler image showing tricuspid regurgitation jet directed towards the interatrial septum (*C*).

**Figure 2:**
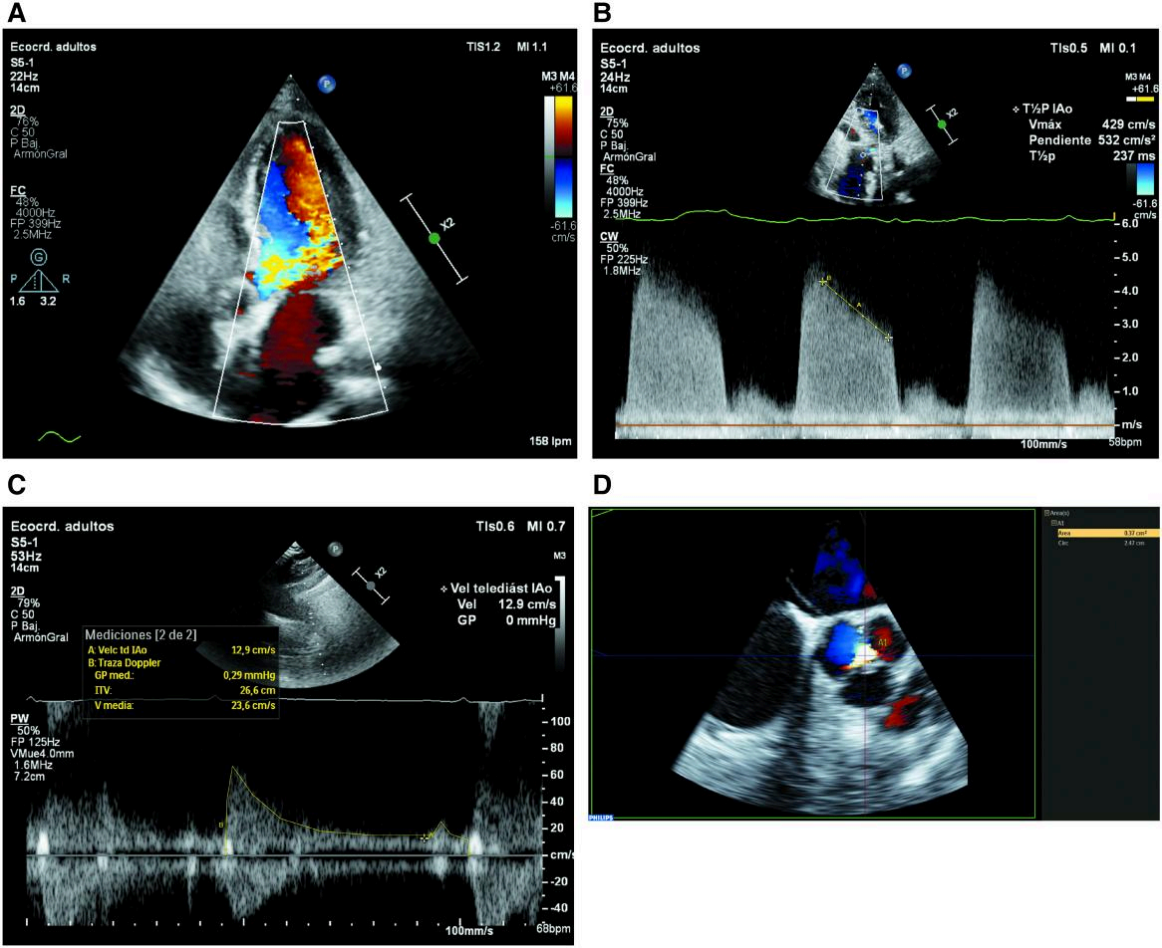
Severe aortic regurgitation. (*A*) Doppler showing the aortic regurgitation jet. (*B*) Continuous-wave Doppler showing a steep slope of the regurgitant jet (PHT < 250 ms). (*C*) Pulsed Doppler with VTI measurement in the descending thoracic aorta (VTI: 26 cm). (*D*) Multiplanar planimetric measurement of the effective regurgitant orifice area (EROA: 37 mm²).

**Figure 3:**
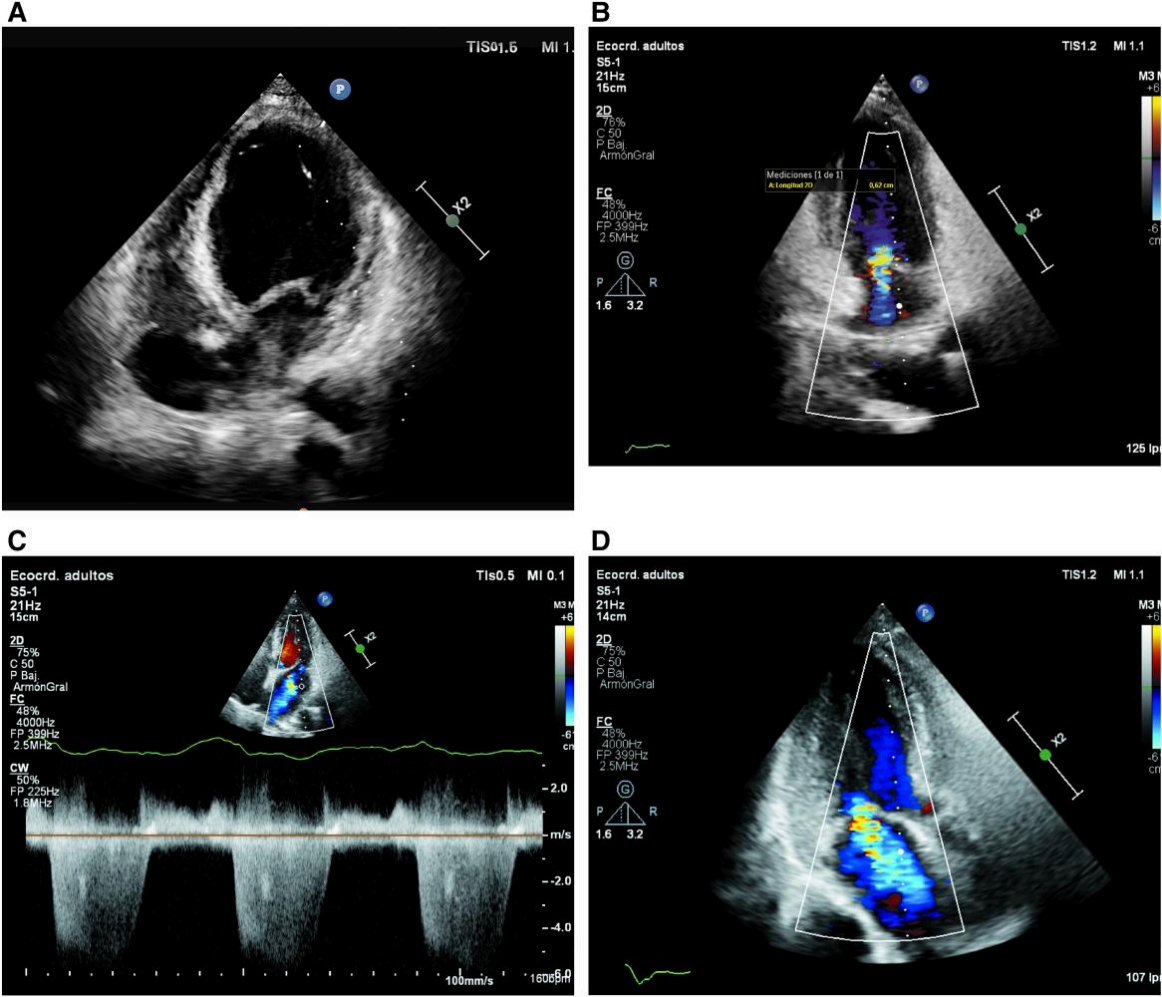
Moderate–severe mitral regurgitation. (*A*) Four-chamber view showing thickening of the mitral valve secondary to carcinoid disease. (*B*) Two-chamber view showing mitral regurgitation with a vena contracta measuring 6 mm. (*C*) Continuous-wave Doppler of mitral regurgitation. (*D*) Doppler image of mitral regurgitation in a three-chamber view.

In January 2024, the patient was referred to our cardiology department by progressive shortness of breath on exertion over a period of two months. B-type pro-natriuretic peptide (NT-proBNP) was mildly elevated (1216 pg/mL, cut-off: 900 pg/mL) and a panfocal systolic murmur was detected. An initial transthoracic echocardiography (TTE) was performed, which revealed extensive multivalvular involvement, affecting the tricuspid, aortic, and mitral valves. The tricuspid valve exhibited rigid leaflets with marked systolic and diastolic restriction (Carpentier type IIIa), severe tricuspid stenosis (mean gradient: 9 mmHg, *Figure 1A*, see Supplementary material online, *Videos S1*, *S3*, and *S6*), and moderate tricuspid regurgitation (VC: 5 mm, *Figure 1B* and *C*, see Supplementary material online, *Videos S1*, *S3*, and *S6*). The aortic and mitral valves were similarly affected, resulting in severe aortic regurgitation (EROA: 37 mm^2^, PHT: 237 mseg, *Figure 2A–D*, see Supplementary material online, *Videos S12*, *S15*, and *S16*) and moderate mitral regurgitation (VC: 6 mm, *Figure 3A–D*, see Supplementary material online, *Videos S9* and *S11*). Loop diuretic therapy was initiated to improve dyspnoea. The symptoms of shortness of breath gradually worsened, and dyspnoea on exertion with mild physical activity severely interfered with her daily life.

We discussed how to formulate a treatment plan with the heart team. Finally, the patient was referred to the cardiac surgery department for evaluation of aortic, mitral, and tricuspid valve replacement. However, in early 2025, the patient was admitted with refractory hypoxemic respiratory failure, requiring non-invasive mechanical ventilation, followed by high-flow oxygen therapy. The chest X-ray at admission was normal. NT-proBNP was slightly elevated (1286 pg/mL) and D-dimer was negative. Inflammatory markers were within normal limits, and viral panel testing was negative. During hospitalization in the Cardiology unit, a preoperative assessment for cardiac surgery was performed, including right and left heart catheterization. Cardiac catheterization revealed no coronary lesions, normal pulmonary capillary wedge pressure, no oxygen saturation step-up, but elevated right atrial pressure (15 mmHg).

**Figure 4:**
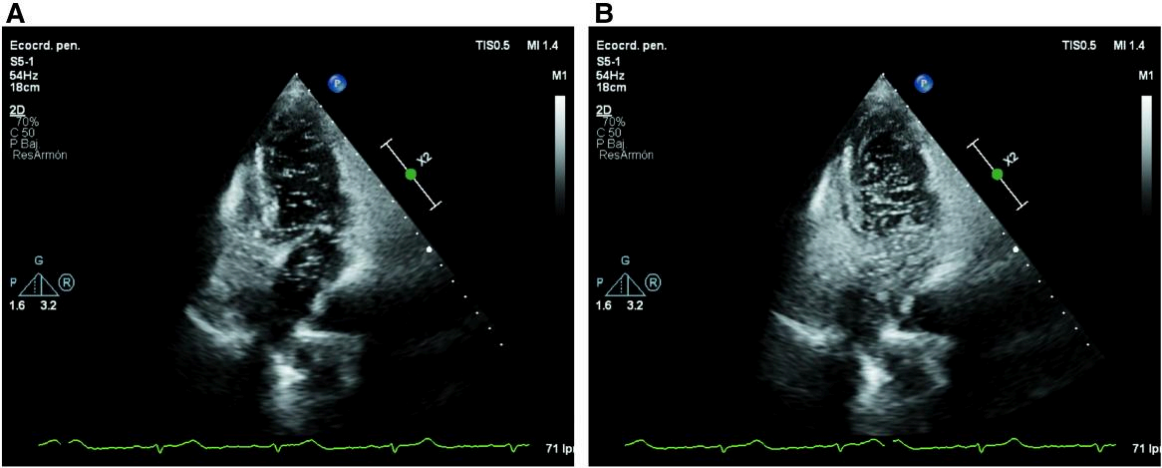
Agitated saline contrast test. Supine position (*A*). Upright position (sitting) (*B*).

**Figure 5:**
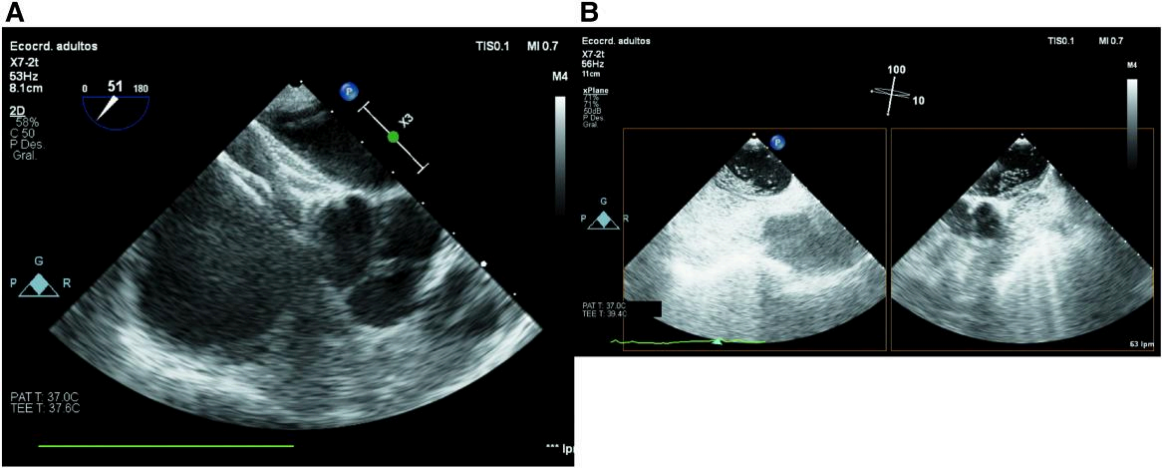
Transoesophageal echocardiography. Amplatzer device position (30 mm) (*A*). Agitated saline contrast test before PFO closure. XPlane view (*B*).

During admission the patient experienced multiple episodes of severe desaturation, particularly with positional changes, which did not adequately improve with high-flow oxygen. Given the unremarkable findings from complementary tests and relentness hypoxia, a right-to-left shunt was suspected. In February 2025, a second TTE with agitated saline contrast was performed. The first administration (decubitus position) revealed a severe shunt within the first three cardiac cycles through a patent foramen ovale (PFO), with a massive shunt observed in the upright position (*Figure 4A* and *B*). A subsequent transoesophageal echocardiogram (TEE) confirmed a high-risk PFO, characterized by an atrial septal aneurysm, a prominent Eustachian valve, an opening >4 mm. The shunt was also confirmed in supine position by nearly identical PaO₂ values (46 and 47 mmHg) after 20 min of oxygen at FiO₂ 50% and 100%. PFO closure was performed under general anaesthesia and guided by TEE using a 30 mm Amplatzer Cribriform Multifenestrated Septal Occluder, with no complications and rapid resolution of hypoxia (*Figure 5A* and *B*). Within 24 h post-procedure, in the intensive care unit, oxygen saturation remained at 100% without supplemental oxygen. However, on the following day, the patient experienced an aspiration event secondary to malposition of the nasogastric tube, resulting in respiratory failure and elevated inflammatory markers. The patient was treated with broad-spectrum antibiotics and invasive mechanical ventilation. Despite medical management, a few days later, the patient suffered a cardiorespiratory arrest and died on the same day, in March 2025.

## Discussion

This report describes an exceptional case of CHD. Carcinoid tumours are rare, and fewer than 10% develop CS.^1^ Normally, vasoactive substances are inactivated in the liver and lungs and patients remain asymptomatic. In this case, the presence of hepatic metastases allowed vasoactive substances to reach the right side of the heart (via hepatic vein), leading to CHD and systemic symptoms. Once CS is established, more than half of affected patients develop CHD, which typically involves right-sided heart valves causing stenosis, regurgitation, or both.^2^ In this case, the tricuspid valve was the only one affected right valve. The pulmonary valve, involved in up to 60% of cases, remained unaffected. CHD is described in the literature as a heterogeneous condition that may present with a wide spectrum of valvular involvement on echocardiography. TTE remains the gold standard for the diagnosis and follow-up of CHD.

Survival in patients with carcinoid tumours has improved with the use of somatostatin analogues and cardiac surgery. Without treatment, the prognosis remains poor, with three-year survival rates ∼30%. Right heart failure remains the leading cause of death.^3^

The mechanism by which heart valves are affected remains unclear but evidence suggests that serotonin plays a key role in the develop of valve fibrosis.^4,5^

Left-sided valve involvement is rare (<10%) and typically requires a right-to-left shunt.^2^ Interestingly, 50% of patients with CHD present with a PFO, a higher prevalence than in the general population. Moreover, studies suggest that PFOs are more frequently detected on follow-up echocardiograms, likely due to progressive right-sided valvular disease.^6,7^ The progression of tricuspid valve disease played a key role in the development of massive right-to-left shunting in this patient. Elevated right atrial pressure and a regurgitant jet directed towards the PFO support this hypothesis. Additionally, TEE revealed a high-risk PFO.

Based on the available literature, the PFO should be systematically evaluated in patients with CS, particularly in those with left-sided valve involvement and hypoxia relentless.^8^ Traditionally, invasive procedures and therapies in cardiology are indicated when the patient’s life expectancy exceeds 12 months. Percutaneous PFO closure is considered a palliative option for hypoxia secondary to a right-to-left shunt; however, its efficacy in patients with severe carcinoid heart disease remains uncertain. In this case, PFO closure was performed as a palliative intervention aimed at improving the patient’s quality of life.^9,10^ The 2022 ESC/EACTS Guidelines on Cardio-Oncology state that interatrial shunt closure should be considered in patients with left-sided carcinoid valve involvement. However, supporting data are limited. The rapid improvement in oxygen saturation following PFO closure supports early intervention in similar clinical scenarios.

Patients with CS may experience dyspnoea due to various causes, including the tumour itself, chemotherapy, and valvular involvement. Valve replacement remains the most effective treatment option for advanced CHD.^11^ This may lead to PFO-related shunting being overlooked as a potential cause. Further studies are needed to assess the role of PFO in patients with CS.

In conclusion, we report an exceptional case of bilateral CHD with a large PFO-related shunt, successfully treated by percutaneous closure. The shunt, rather than the valvular disease, proved to be the main issue in this patient. To our knowledge, the combination of significant left-sided valvular involvement and severe tricuspid stenosis makes this an unusual presentation of CHD.

## Lead author biography


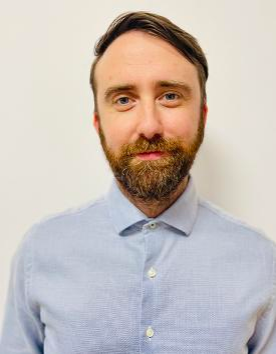


Javier Bertolín Boronat was born in Valencia in 1987. His deep passion for science and a long standing medical tradition in his family led him to study medicine, graduating from the University of Valencia in 2013. He currently works as a staff cardiologist at Arnau de Vilanova Hospital in Valencia, Spain, where he is part of the Cardiac Imaging Unit.

## Supplementary Material

ytaf444_Supplementary_Data

## Data Availability

The data underlying this article are available in the article and online Supplementary material.
